# Evaluation of Antibiotic Sensitivity in Methicillin-Resistant Staphylococcus aureus Isolates From Clinical Specimens in a Tertiary Care Hospital

**DOI:** 10.7759/cureus.72628

**Published:** 2024-10-29

**Authors:** Payal P Patil, Harsha V Patil, Satish Patil

**Affiliations:** 1 Department of Microbiology, Krishna Institute of Medical Sciences, Krishna Vishwa Vidyapeeth (Deemed to Be University), Karad, IND

**Keywords:** antibiotic susceptibility, clinical isolates, clinical laboratory standard institute, methicillin-resistant staphylococcus aureus, β-lactams

## Abstract

Background: Common Gram-positive bacteria *Staphylococcus aureus* can cause infections ranging from minor skin conditions to serious illnesses like sepsis.Methicillin-resistant *S. aureus* (MRSA) emerged in the 1960s and now causes over 50% of hospital infections. In India, MRSA prevalence ranges from 25% in the west to 50% in the south. Contributing factors include prolonged hospital stays and indiscriminate antibiotic use. This study investigates the prevalence and antibiotic resistance patterns of MRSA in clinical isolates from a hospital in Karad. MRSA’s resistance to multiple antibiotics, including vancomycin, poses significant treatment challenges, with limited therapeutic options such as vancomycin, linezolid, daptomycin, and teicoplanin remaining effective. The antibiotics mentioned provide a broader context for understanding MRSA resistance. In this study, only those specifically relevant to the hospital's data have been included in detail in the result table antibiogram of MRSA.

Material and methods: A two-year laboratory-based analysis of MRSA strains in a hospital that provides tertiary care was conducted to assess their antibiotic susceptibility profiles.

Results: In all, 100 isolates of MRSA were examined for patterns of antibiotic susceptibility in various clinical specimens. Among these 41 (41%) were from pus samples, 18 (18%) from wound swabs/discharge, 15 (15%) from urine, 13 (13%) from blood, 4% from sputum, and 3% from tips were found to have smaller percentages than umbilical swabs (2 (2%)) and tissue (1 (1%)) and vaginal swabs( 1 (1%)) and sinus tract samples (1 (1%)). The frequency of MRSA strains was more in males (59%). Linezolid showed the highest sensitivity at 86%.

Conclusion: This study highlights the growing challenge of MRSA and other resistant bacteria in hospital settings, complicating treatment choices. Effective MRSA​​​​​​​ management requires stringent antibiotic policy, infection control procedures, and thorough susceptibility testing. These strategies are critical to preserving viable treatment options and combating antibiotic resistance in healthcare environments.

## Introduction

The widespread Gram-positive bacterium *Staphylococcus aureus* is the cause which includes a broad variety of disorders, from simple skin infections to serious situations like endocarditis, pneumonia, and sepsis. It has a big impact on infections that are acquired in hospitals and also in the community [1]. A major worry that first surfaced in the late 1960s is that S. *aureus* is now accountable for over 50% of hospital-acquired infections due to resistance to methicillin [2]. Two factors contribute to the spread of this infection: prolonged hospital stays, improper use of antibiotics, and lack of awareness [3]. With regional incidence rates ranging from 25% in the western areas to 50% in the southern regions, methicillin-resistant *S. aureus *(MRSA) has become endemic throughout India [4]. MRSA is genetically adapted to the degree that it poses considerable resistance to a greater variety of antibiotics like β-lactams [5]. MRSA infections have recently become the focus of intense media attention. In 2005, the United States press described MRSA as the "superbug," because it killed more people than the acquired immunodeficiency syndrome (AIDS) did [6]. MRSA has been reported to exhibit complete resistance to gentamicin, erythromycin, penicillin, ceftriaxone, and ampicillin in Trinidad and Tobago [7]. Only higher-cost drugs like vancomycin, linezolid, teicoplanin, daptomycin, and streptogramins are available as treatment options, and there are cases of resistance developing to vancomycin, although infrequent, having been documented [8].

In this study, we aim to examine and report the prevalence of MRSA with its resistance to various clinically relevant drugs in the clinical *S. aureus* isolates collected from various clinical samples in tertiary care hospital Karad.

## Materials and methods

This research was conducted in the Microbiology Department of Krishna Institute of Medical Sciences (Deemed to Be University), Karad, India, following ethical approval protocol number 073/2021/2022, for the period from November 2022 to November 2023. Samples were collected from patients across a range of ages and genders, including pus, sputum, wound swabs, blood, urine, and other body fluids.

Direct Gram staining was performed on the sample smears, and the samples were cultured on nutrient agar, blood agar, and Mannitol salt agar, followed by incubation for 24 hours at 37°C. Bacterial colonies were identified based on their morphology, Gram stain, catalase, and coagulase tests, along with biochemical reactions, as per Koneman’s protocols [9].

Phenotypic test for* S. aureus*: detection of methicillin-resistant strain

MRSA Detection Test

**Figure 1:**
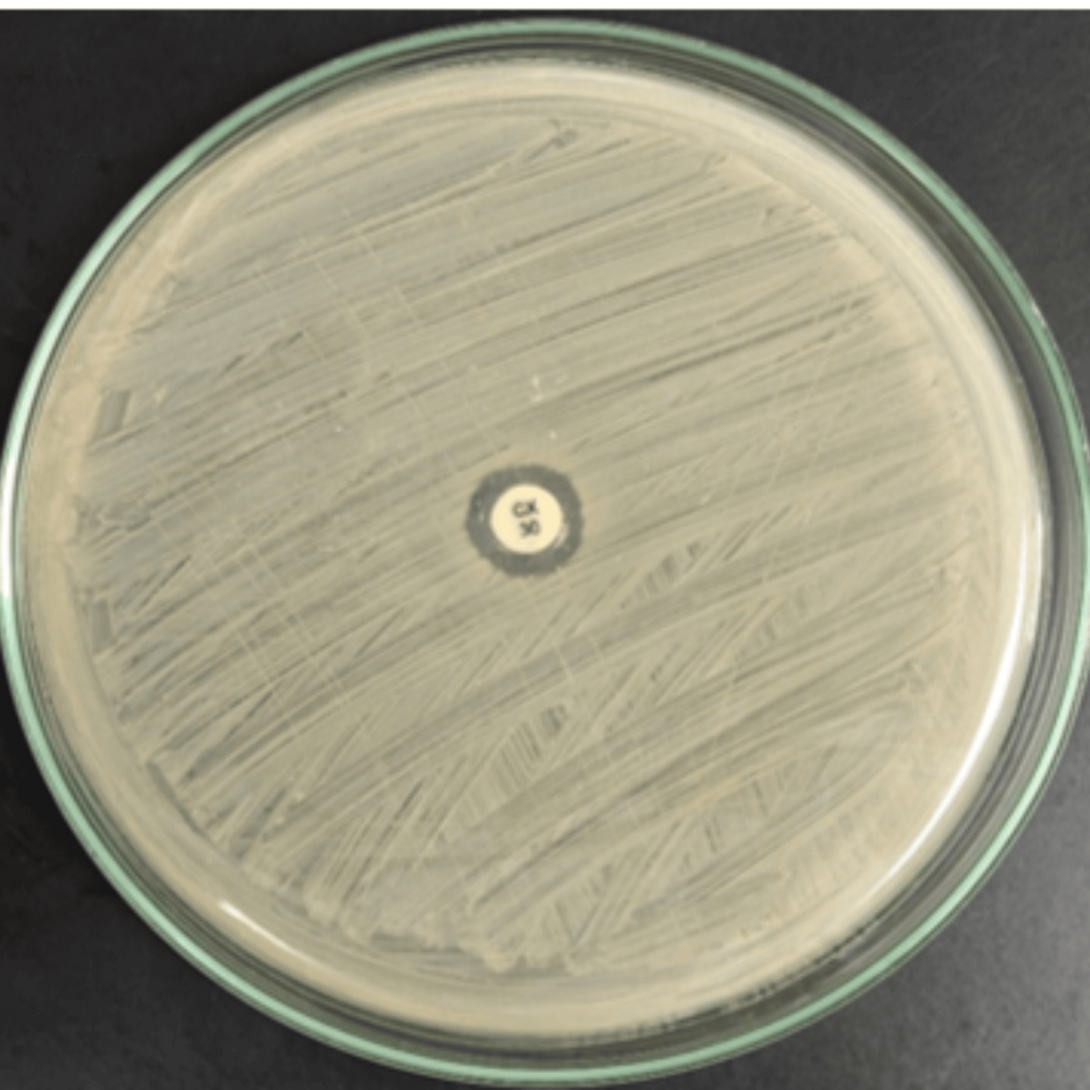
Cefoxitin disc (30 µg) diffusion method for detection of methicillin-resistant Staphylococcus aureus

After placing 30 µg cefoxitin discs on Muller-Hinton agar, incubation of the plates lasted for 24 hours at 37°C. As shown in (Figure 1), zones of inhibition were measured, with a diameter of 22 mm or greater indicating methicillin susceptibility, and 21 mm or less indicating resistance. *S. aureus* American Type Culture Collection (ATCC) 25923 was used as the control strain for quality assurance.

Antimicrobial Susceptibility Test

Antibiotic susceptibility testing was conducted using the Kirby-Bauer Disk Diffusion method, following Clinical and Laboratory Standards Institute (CLSI, 2022) guidelines [10]. The antibiotics tested included penicillin (10 µg), methicillin (5 µg), gentamicin (10 µg), ampicillin (10 µg), nitrofurantoin (100 µg), co-trimoxazole (25 µg), amikacin (30 µg), clindamycin (2 µg), erythromycin (15 µg), ciprofloxacin (5 µg), and levofloxacin (5 µg). For vancomycin (30 µg) and linezolid (30 µg), the testing followed the protocol specified by Kumar et al. [11].

**Figure 2:**
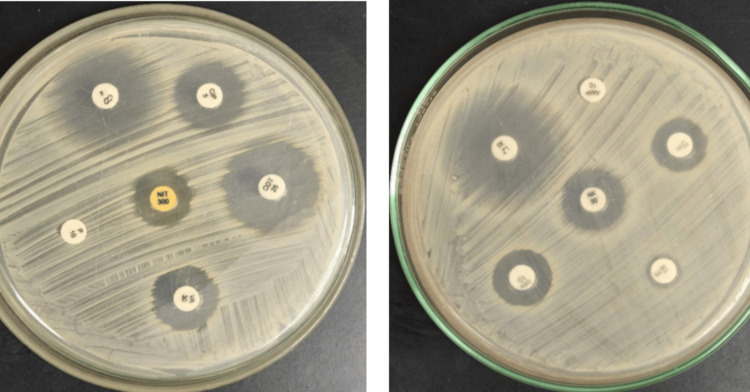
Antibiotic sensitivity testing

For antimicrobial susceptibility testing, Muller Hinton agar was used. As a control for this test, the ATCC *S. aureus* 25923 strain was used (Figure 2).

## Results

From various clinical samples, 100 isolates of *S. aureus* that were clinically important were recovered in pure form. The antimicrobial sensitivity of 100 MRSA isolates to 11 antibiotics is shown in Table 1. At 86%, linezolid showed the highest sensitivity, followed by co-trimoxazole at 59% and nitrofurantoin at 84%. Clindamycin and vancomycin each had a sensitivity of 44%, whereas gentamicin and amikacin demonstrated a sensitivity of 53%. Less sensitive antibiotics were levofloxacin (14%), ciprofloxacin (16%), and erythromycin (23%). Levofloxacin exhibited resistance at 86%, ciprofloxacin at 84%, and penicillin at 100%. About 77% of patients were resistant to erythromycin and clindamycin, compared to 56% to vancomycin and gentamicin. Nitrofurantoin showed 41% resistance, amikacin, and co-trimoxazole 47% resistance. At 16%, linezolid had the lowest resistance rate (Table 1).

**Table 1:** Antibiogram of methicillin-resistant Staphylococcus aureus

Antibiotics	Methicillin-resistant *Staphylococcus aureus *(MRSA)	
	Sensitive (%)	Resistant (%)
Penicillin-G	0	100 (100%)
Erythromycin	23 (23%)	77 (77%)
Gentamycin	53 (53%)	47 (47%)
Co-trimoxazole	59 (59%)	41 (41%)
Clindamycin	44 (44%)	56 (56%)
Levofloxacin	14 (14%)	86 (86%)
Ciprofloxacin	16 (16%)	84 (84%)
Amikacin	53 (53%)	47 (47%)
Nitrofurantoin	84 (84%)	16 (16%)
Linezolid	86 (86%)	14 (14%)
Vancomycin	44 (44%)	56 (56%)

## Discussion

The recent rise in MRSA and other multidrug-resistant strains in large hospitals posed a significant challenge in selecting appropriate antimicrobial treatments. These resistant strains burdened healthcare resources and contributed to high morbidity and mortality rates among patients. Swift and accurate detection of methicillin resistance in *Staphylococci *was crucial. It helped in choosing effective antibiotic therapy, controlling MRSA spread, implementing timely infection control measures, and preserving antibiotic efficacy for sustainable healthcare practices.

In the present study, 100 MRSA isolates were analyzed, revealing that individuals aged 21-40 exhibited the highest frequency, accounting for 73.47% of cases. This is higher compared to the study by Goyal et al. [12], which reported 51% of MRSAcases in individuals aged 20-40 years. Additionally, 59% of cases were observed in males, whereas females reported 41%. These findings compared with Lohan et al. [13], who reported a higher prevalence of MRSA​​​​​​​ among males (67.9%) compared to females (32.1%). Regarding the types of clinical samples, pus samples were the predominant source of *S. aureus* isolates, constituting 41% of the total. This study compares with Pfingsten-Würzburg et al. [14], study who observed that 47% of *S. aureus* isolates came from pus, and Kumar et al. [11], who reported 31.45% of MRSA​​​​​​​ isolates from pus samples.

In the current research, when the sensitivity of MRSA​​​​​​​ isolates was evaluated, it was discovered that the most potent antimicrobial drug was linezolid, which had an 86% sensitivity rate. Levofloxacin (86%) showed the highest level of resistance. The outcomes are in line with the research done by Goyal et al. [12] stated that every MRSA isolate in their investigation (100%) was sensitive to both vancomycin and linezolid and showed resistance to ciprofloxacin 58.05%, and erythromycin 79.75%, highlighting the continued effectiveness of these antibiotics against MRSA*.* Similar findings were reported by Basavaraj [15] revealed that while linezolid had a high sensitivity rate of 89.3%, MRSA​​​​​​​ isolates showed significant resistance to ciprofloxacin (76.1%) and erythromycin (64.0%).

Strengths

Comprehensive susceptibility testing and rapid laboratory diagnostics are crucial tools that enable clinicians to effectively manage MRSA​​​​​​​ infections, ensuring targeted and appropriate treatment.

Limitations

The increasing prevalence of MRSA complicates the selection of effective antimicrobial therapies, necessitating the implementation of robust antibiotic policies and stringent infection control measures. Also, the reliance on existing testing methods may delay the identification of resistant strains, underscoring the need for ongoing advancements in diagnostic capabilities to preserve effective treatment options in healthcare settings.

## Conclusions

The increasing occurrence of multidrug-resistant strains of *S. aureus* and other antibiotic-resistant bacteria in hospital environments has made it difficult to choose the best antimicrobial therapies for related infections. Therefore, implementing strong antibiotic policies and closely following infection control protocols are essential actions to reduce the spread of resistance isolates. A thorough susceptibility test and other rapid and reliable laboratory diagnostics are essential for efficiently managing, treating, and preventing MRSA infections. These tactics are crucial for preserving therapeutic modalities that work and reducing the effects of antibiotic resistance in healthcare settings.
